# Supplementary figures and images for: RACK1 MARylation regulates translation and stress granules in ovarian cancer cells
Source: J Cell Biol. 2025 Jan 6;224(2):e202401101. doi: 10.1083/jcb.202401101 (PMC11702359; doi:10.1083/jcb.202401101)

Fig. 1 - Challa *et al.* (2024) - Raw Data

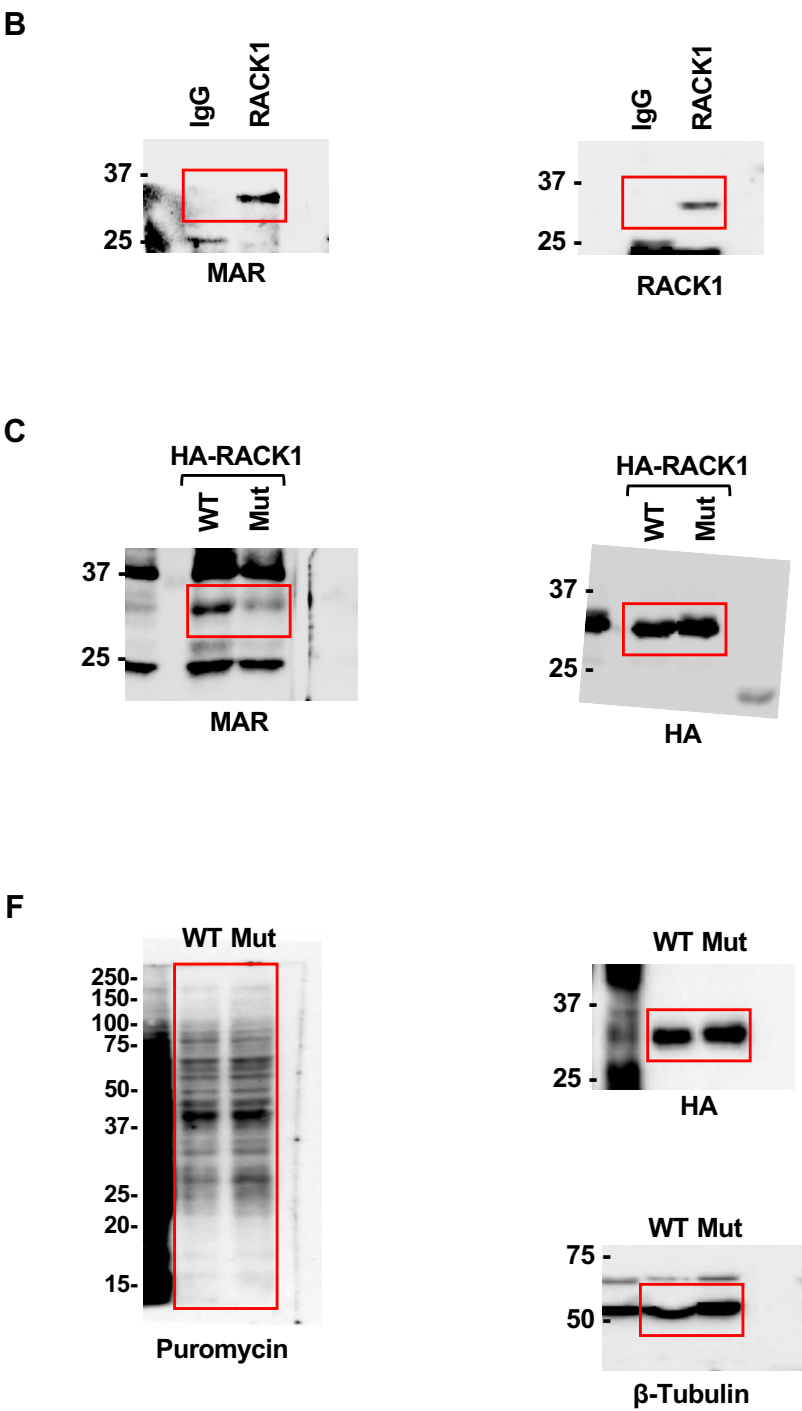

Supplement: SourceData F1 — is the source file for Fig. 1. [file jcb_202401101_sourcedataf1.pdf]

Fig. 2 - Challa *et al.* (2024) - Raw Data

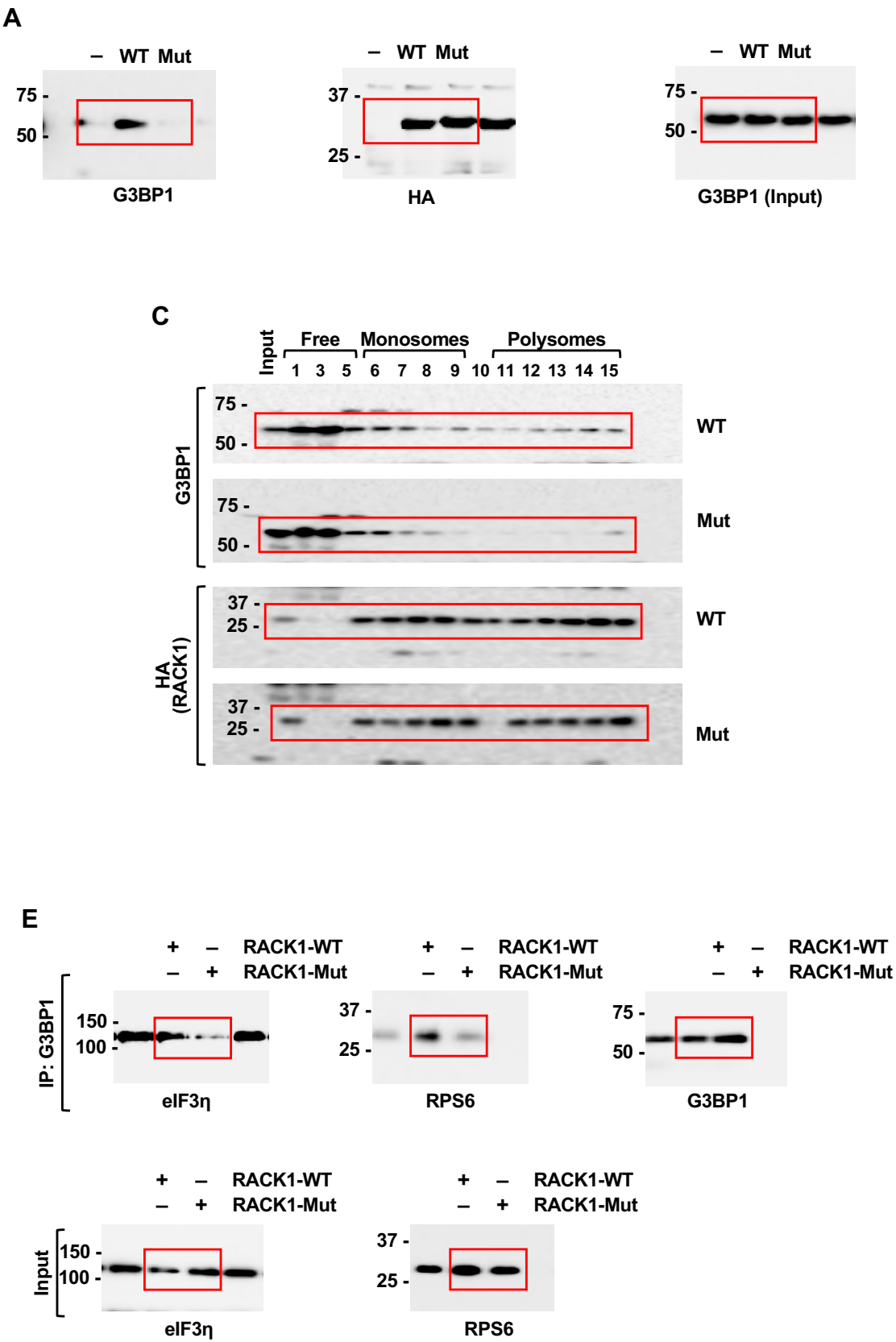

Supplement: SourceData F2 — is the source file for Fig. 2. [file jcb_202401101_sourcedataf2.pdf]

Fig. 3 - Challa *et al.* (2024) - Raw Data

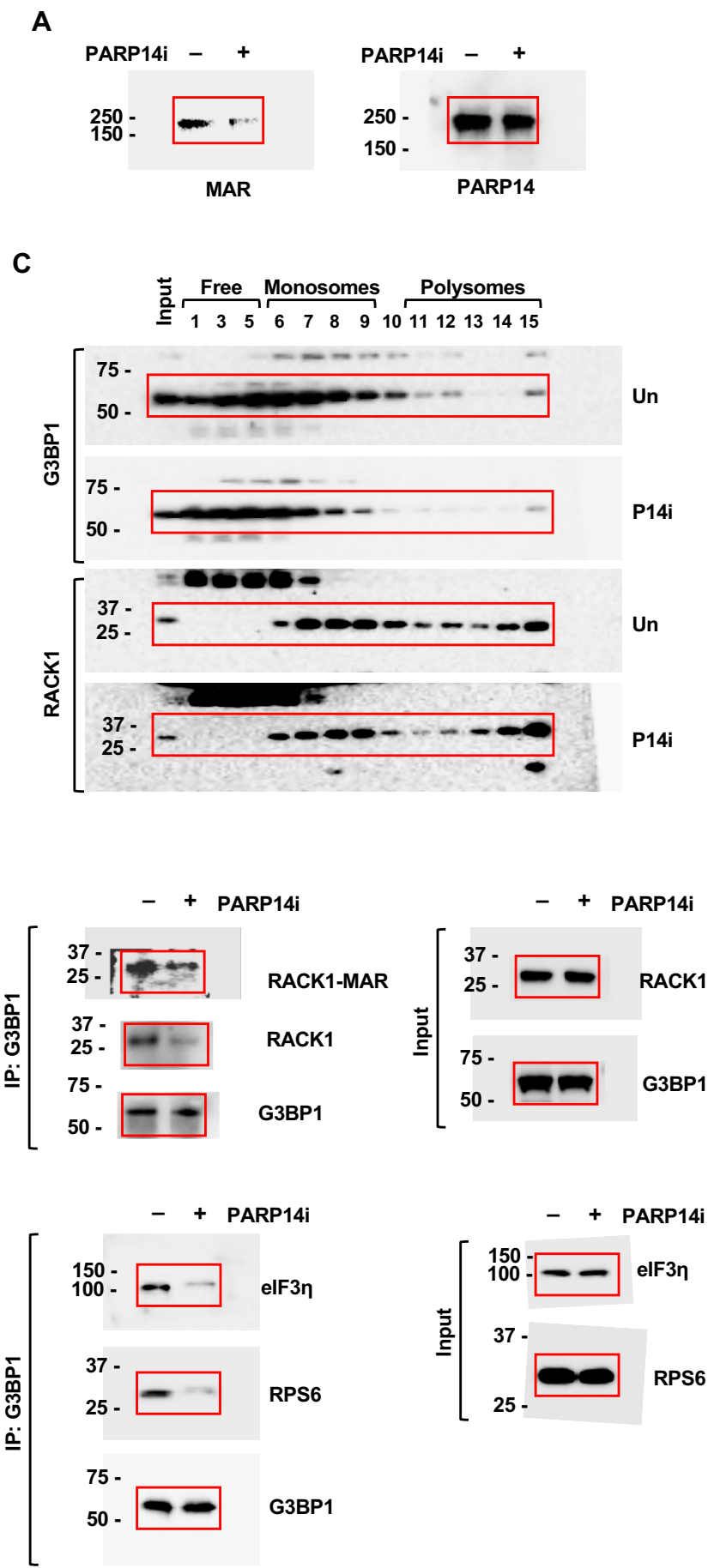

Supplement: SourceData F3 — is the source file for Fig. 3. [file jcb_202401101_sourcedataf3.pdf]

Fig. 5 - Challa *et al.* (2024) - Raw Data

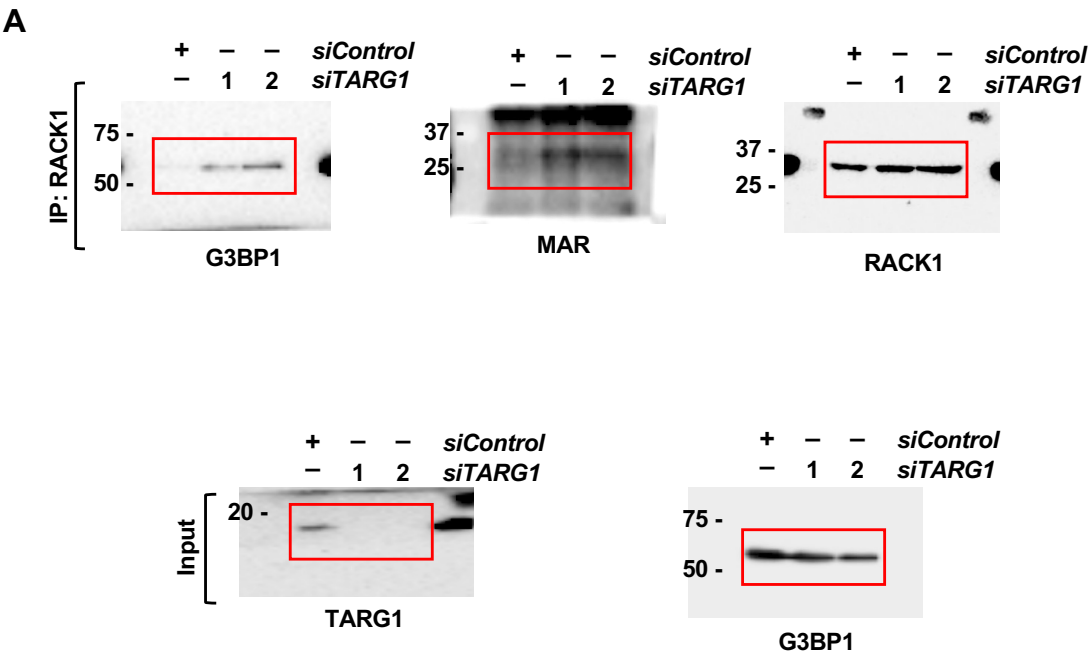

Supplement: SourceData F5 — is the source file for Fig. 5. [file jcb_202401101_sourcedataf5.pdf]

Fig. 6 - Challa *et al.* (2024) - Raw Data

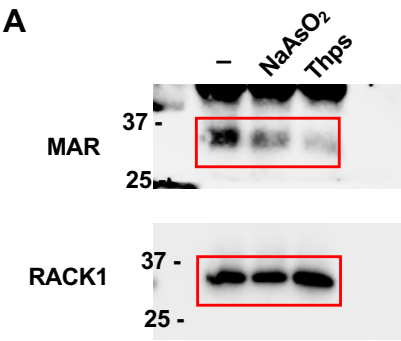

Supplement: SourceData F6 — is the source file for Fig. 6. [file jcb_202401101_sourcedataf6.pdf]

Suppl. Fig. 1 - Challa *et al.* (2024) - Raw Data

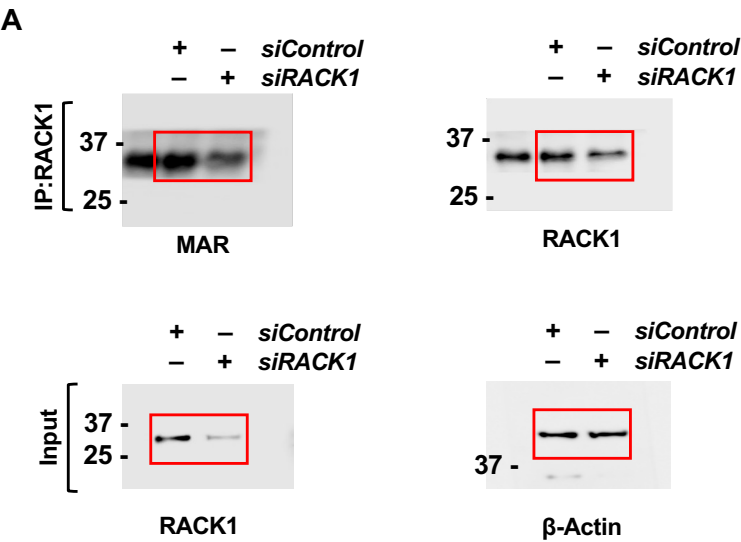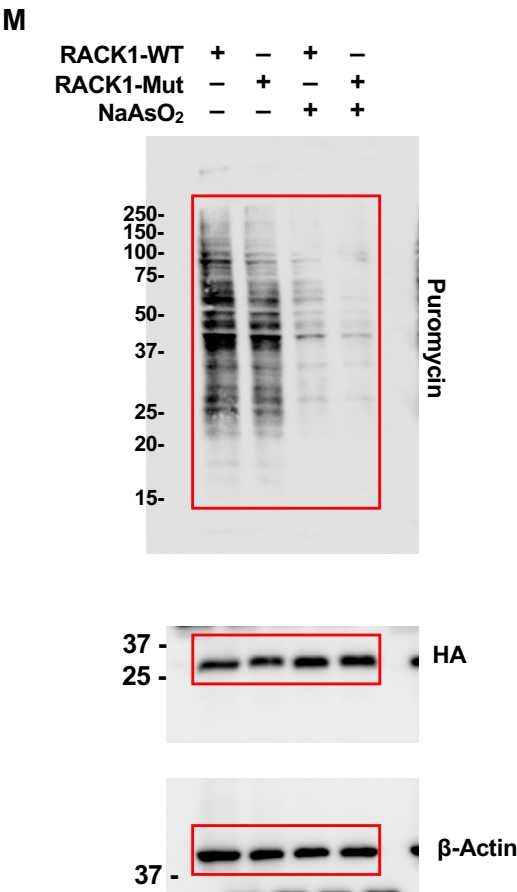

Supplement: SourceData FS1 — is the source file for Fig. S1. [file jcb_202401101_sourcedatafs1.pdf]

Suppl. Fig. 3 - Challa *et al.* (2024) - Raw Data

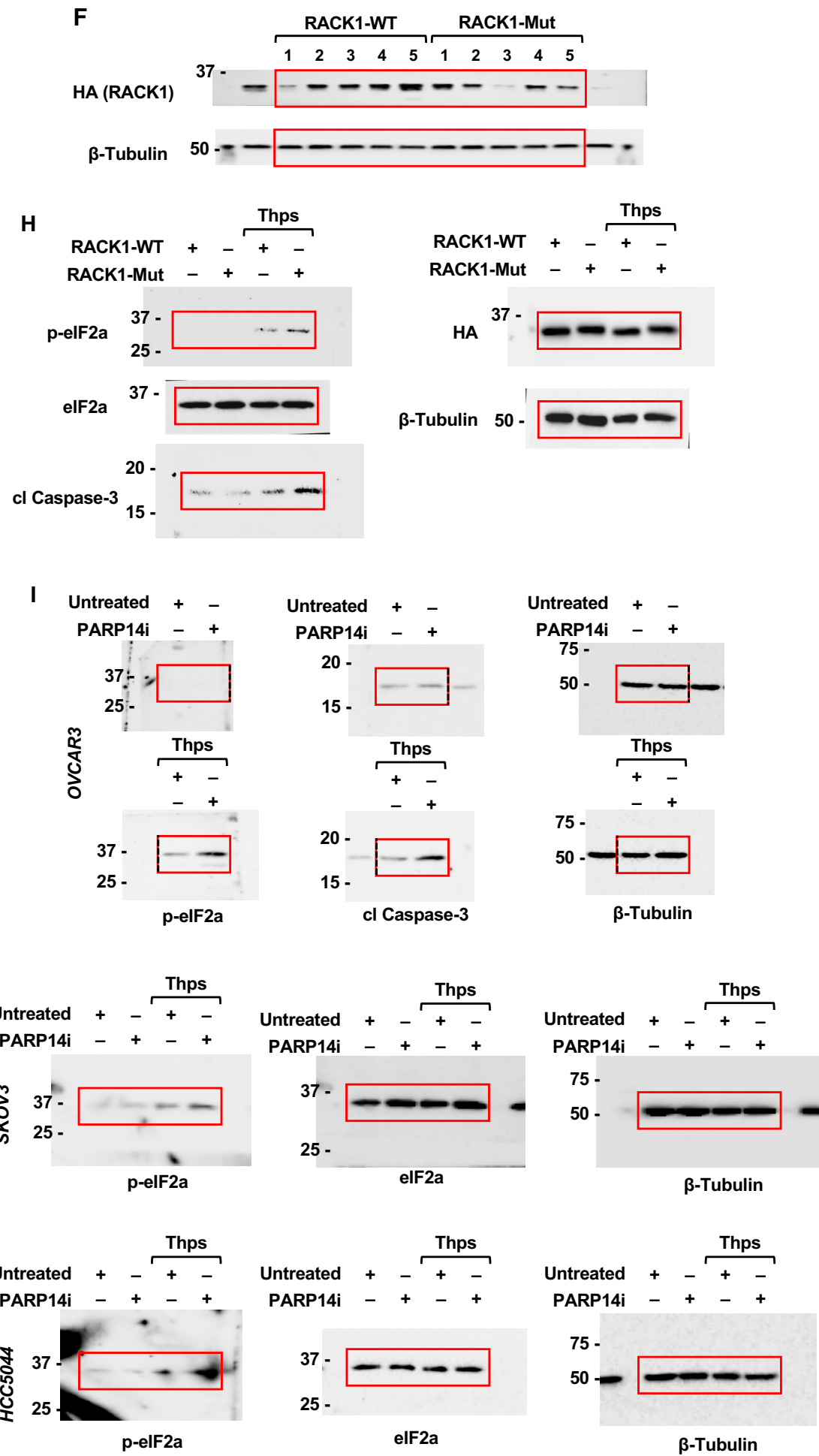

Supplement: SourceData FS3 — is the source file for Fig. S3. [file jcb_202401101_sourcedatafs3.pdf]

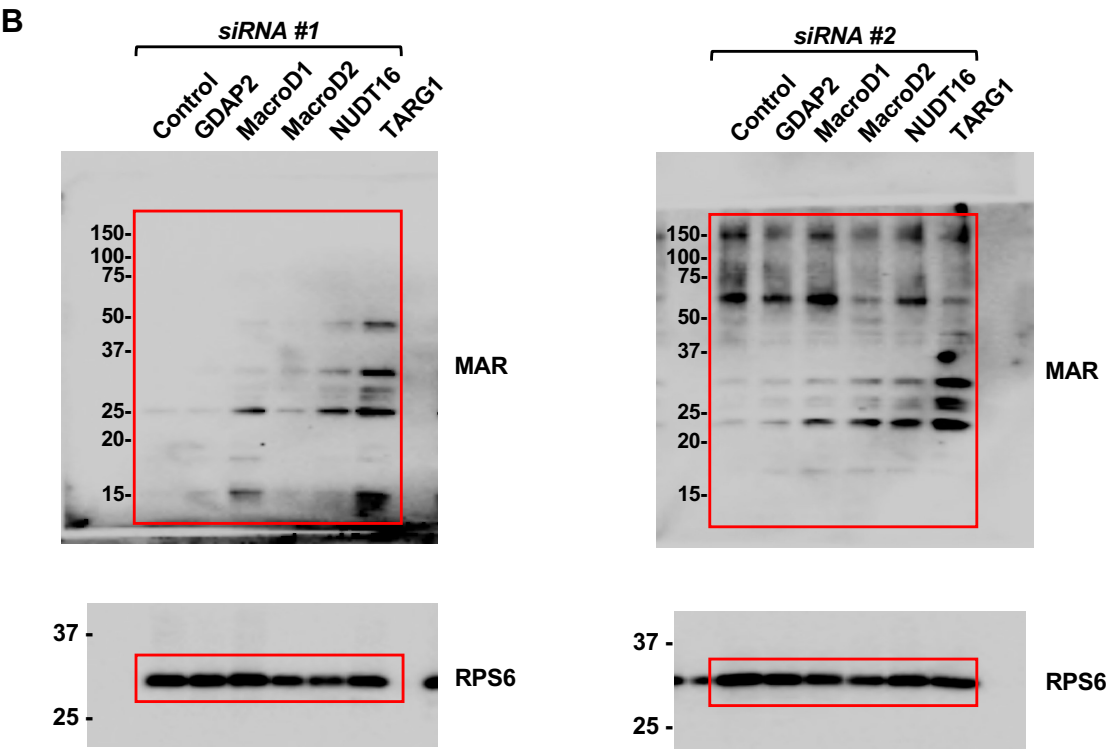

Supplement: SourceData FS4 — is the source file for Fig. S4. [file jcb_202401101_sourcedatafs4.pdf]
